# Effect of Dala‐Led Behavior Change Communication on Complementary Feeding Practices in Infants and Young Children: A Cluster‐Randomized Controlled Trial in Agro‐Pastoral Settings of Afar Region, Ethiopia

**DOI:** 10.1111/mcn.70254

**Published:** 2026-09-23

**Authors:** Anwar Seid Ali, Tefera Belachew, Shemsu Kedir, Kalkidan Hassen Abate

**Affiliations:** ^1^ Department of Human Nutrition & Dietetics Faculty of Public health, Institute of Health Jimma University Jimma Oromia Region Ethiopia; ^2^ Departments of Nursing College of Medical and Health Sciences Samara University Semera Afar Region Ethiopia; ^3^ Departments of Public Health College of Medicine and Health Sciences Werabe University Werabe Central Ethiopia Region Ethiopia

**Keywords:** agro‐pastoralist, behavior change communication, community‐based intervention, complementary feeding, Ethiopia, infant and young child feeding

## Abstract

Suboptimal complementary feeding remains a major public health concern in Ethiopia, particularly among pastoralist and agro‐pastoral communities where mobile livelihoods hinder access to conventional health services. In these settings, numerous studies have highlighted the potential of leveraging culturally embedded leadership structures to enhance health promotion efforts. This study examined whether engaging traditional Dala leaders as agents of Nutrition Behavior Change Communication (DALA‐BCC) could improve complementary feeding indicators among infants and young children in the agro‐pastoral areas of northern Ethiopia. A cluster‐randomized controlled trial was conducted in Dubti District, Afar Region, involving 16 non‐adjacent clusters randomly assigned to intervention and control arms (eight each). A total of 474 mother–infant pairs (237 per arm) were enrolled at 6–17 months of age and followed over 6 months. Over a 6‐month period, the intervention group received DALA‐BCC delivered by Dala leaders, consisting of monthly group sessions and cooking demonstrations during the first 3 months, followed by home‐based individualized counseling from Month 3 to Month 6, while the control group continued usual care. Core infant and young child feeding (IYCF) indicators were measured at baseline and endline. At end line, the intervention group achieved higher rates of minimum dietary diversity (MDD) (39.7% vs. 24.7%; *p* = 0.001), minimum meal frequency (MMF) (67.9% vs. 47.6%; *p* < 0.001), and minimum acceptable diet (MAD) (28.6% vs. 12.1%; *p* < 0.001). Generalized estimating equation (GEE) analysis showed significant time × group interactions, indicating greater improvements in the intervention group for MDD (RR = 2.43; 95% CI: 1.35–4.39), MMF (RR = 2.08; 95% CI: 1.25–3.48), and MAD (RR = 2.35; 95% CI: 1.10–5.04). The findings demonstrate that the DALA‐BCC intervention significantly improved complementary feeding practices, highlighting the effectiveness of culturally grounded, community‐led nutrition strategies in Agro‐pastoral settings.

**Trial Registration:**

PACTR202311816542918. Registered 10 November 2023.

## Summary

- Suboptimal complementary feeding remains a major public health concern in pastoralist and agro‐pastoral communities of Ethiopia due to mobile livelihoods and limited access to health services.
- Engaging traditional Dala leaders to deliver Nutrition Behavior Change Communication (DALA‐BCC) improved key complementary feeding practices, including dietary diversity and meal frequency.
- The intervention was well‐received and demonstrated the potential of culturally grounded, community‐led approaches to enhance infant and young child feeding practices.
- Future programs should consider integrating traditional leadership structures and extending follow‐up periods to evaluate long‐term impacts on child nutrition outcomes.

## 1 Introduction

Suboptimal dietary practices among infants and young children remain a major public health concern, with many failing to meet minimum standards for dietary diversity, meal frequency, or overall diet adequacy (WHO and UNICEF 2021; UNICEF 2023). Complementary feeding practices remain suboptimal across many low‐ and middle‐income countries. Globally, only about 65% of infants aged 6–8 months are introduced to solid, semi‐solid, or soft foods as recommended. The situation is particularly concerning in West and Central Africa, where coverage rates fall as low as 45%, highlighting persistent gaps in early childhood nutrition (UNICEF 2023). Only about 29% of children aged 6–23 months meet the minimum dietary diversity (MDD) standard, with particularly low coverage in South Asia (approximately 23%) and sub‐Saharan Africa (around 20%). Even more concerning, the minimum acceptable diet (MAD) which integrates both dietary diversity and meal frequency is achieved by just 20% of children worldwide, highlighting critical gaps in complementary feeding across low‐resource settings (UNICEF 2023). These deficiencies contribute to widespread undernutrition, stunting, and other adverse health outcomes, particularly in low‐resource settings (UNICEF and W 2023).

Socioeconomic factors such as poverty, food insecurity, and limited access to diverse, nutrient‐rich foods are major drivers of suboptimal dietary practices (Marphatia et al. 2016; Dhami et al. 2021; UNICEF 2023; UNICEF and W 2023; Sanghvi et al. 2025). Inadequate maternal knowledge further exacerbates poor dietary diversity and meal frequency (Marphatia et al. 2016; Dhami et al. 2021; Tahreem et al. 2024; Sanghvi et al. 2025). Complementary feeding defined as the timely introduction of solid or semi‐solid foods alongside continued breastfeeding at 6 months varies across livelihood contexts, including pastoralist, agrarian, urban, and rural settings (EPHI and ICF 2019; Ayele et al. 2025; Baffa et al. 2025). These differences reflect variations in socioeconomic status, healthcare access, and household food security, all of which shape infant and young child feeding (IYCF) practices.

In Ethiopia, urban children consistently outperform rural counterparts in achieving MDD, MMF, and MAD (Ayele et al. 2025; Baffa et al. 2025). Inequality is pronounced in pastoralist regions: while 45% of children in Addis Ababa achieve MDD, only 13% in Afar and 9% in Somali meet this standard (EPHI and ICF 2019). The urban advantage likely stems from better market access, higher incomes, and greater exposure to nutrition education. By contrast, pastoralist households face compounded barriers to optimal child nutrition. Their mobile livelihoods often disrupt consistent healthcare access, while livestock‐based diets limit dietary diversity and nutrient intake for young children (Sadler and Catley 2009; Mengistu et al. 2017; Abas et al. 2020).

Given the strong link between nutrition and livelihood contexts, community‐based interventions addressing underlying drivers of malnutrition are essential (Shekar 2016). Evidence from low‐ and middle‐income countries indicates that behavior change interventions delivered through trusted community leaders can improve complementary feeding (Bhandari et al. 2004; Waswa et al. 2015; Kuchenbecker 2017; Mistry et al. 2019; Abiyu and Belachew 2020a; Wild et al. 2020; Rahman et al. 2022; Gebretsadik et al. 2025). However, pastoralist populations remain underrepresented in such research. In Ethiopia, platforms like the women development army (WDA) have improved nutrition in agrarian areas (FMOH 2021), but their reach in pastoralist regions such as Afar is limited due to household mobility. In contrast, Afar communities rely heavily on traditional leadership structures particularly the *Dala* who are deeply trusted and play central roles in community decision‐making. Leveraging these systems may offer a more culturally anchored and sustainable avenue for improving IYCF practices.

In many settings, nutrition interventions are implemented without the guidance of established behavioral theories, limiting their effectiveness and sustainability. The health belief model (HBM) provides a valuable framework for designing culturally sensitive interventions where beliefs strongly influence caregiving practices. The model includes constructs such as perceived susceptibility, severity, benefits, barriers, self‐efficacy, and cues to action factors explaining why caregivers may or may not adopt recommended IYCF behaviors (Tariku et al. 2015). In Ethiopia, HBM‐informed interventions have demonstrated improvements in maternal knowledge, dietary practices, and child nutrition outcomes by addressing belief systems and behavioral obstacles (Tariku et al. 2015; Mulualem et al. 2016; Diddana et al. 2018; Beressa et al. 2024; Riazi et al. 2024). Embedding HBM‐informed behavior change communication (BCC) within indigenous community structures such as the *Dala* could enhance cultural relevance, community acceptance, and sustainability of nutrition programs. Aligning health messaging with trusted local leadership may foster lasting improvements in IYCF practices.

Despite this potential, evidence on the impact of delivering BCC through traditional leadership structures particularly on complementary feeding outcomes such as MDD, MMF, and MAD remains limited in pastoralist settings. To address this gap, this study evaluated the effectiveness of Nutrition Behavior Change Communication (DALA‐BCC) delivered by *Dala* leaders in improving complementary feeding practices among infants and young children in the agro‐pastoralist communities of Afar, Ethiopia.

## 2 Methods

### 2.1 Study Design, Setting and Period

A community‐based, two‐arm parallel‐group cluster randomized controlled trial was conducted among mothers of infants aged 6–17 months at enrollment, residing in the agro‐pastoral communities of Awsi Rasu Zone, Afar Region, Ethiopia. The trial is reported in accordance with CONSORT 2010 extension for cluster randomized trials (Campbell et al. 2012).

The study site is centered around Asayita, the zonal capital, located approximately 628 km northeast of Addis Ababa. Of the nine districts in Awsi Rasu, four are agro‐pastoral, and Dubti district was selected by lottery as the study site. Dubti benefits from irrigated subsistence farming along the Awash River, with maize and vegetables (tomatoes, peppers, onions, leafy greens) being the main crops (CSA 2007). In 2022, the zone's population was estimated at 703,531, including 324,722 women, 80,203 children under 5 years, and 26,313 children under 2 years (CSA 2022).

Although administratively classified as agro‐pastoral, the population maintains strong pastoralist characteristics, with livestock rearing remaining central to livelihoods and household food Security. Health infrastructure in Dubti district includes one referral hospital, three health centers, and ten health posts, while the wider zone is served by three hospitals, 26 health centers, and 108 health posts (RHB 2024). The study was conducted from October 2023 to May 2024.

Within the cultural fabric of Afar communities, traditional structures such as the Dala play a central role in social organization and governance. A Dala comprises a cluster of neighboring households within the same settlement, typically bound by kinship ties, shared interests, or geographic proximity. The size and composition of each Dala vary according to local context and household density. Leadership is vested in a Dala leader often a respected elder or a designated younger successor who serves as the primary decision‐maker and gatekeeper for the group. These leaders oversee a wide range of responsibilities, including dispute resolution, resource management, coordination of communal activities, and stewardship of social and cultural affairs. Their authority and influence are deeply rooted in community trust, comparable to that of clan heads or religious figures, making them pivotal actors in any community‐driven initiative. This culturally grounded approach formed the basis for the design and implementation of the current intervention trial.

The term Dala is specific to the Afar context; however, comparable kinship‐based social organizational structures exist across other pastoralist regions of Ethiopia under different local names. These structures similarly function as key units for social organization, resource sharing, and community‐level decision‐making, thereby enhancing the potential transferability of findings to other pastoralist settings.

These Dala units were selected as the unit of cluster randomization due to their strong social cohesion and functional role in community decision‐making.

### 2.2 Study Population, Eligibility Criteria, and Sample Size Determination

The source population comprised all mothers or primary caregivers of infants aged 6–17 months residing in the agro‐pastoral communities of Awsi Rasu Zone, Afar Region, at the time of the baseline survey. The study population was drawn from caregivers living within selected Dala clusters, while the sample population included those meeting the age eligibility criteria within these clusters. Inclusion criteria required participants to have a singleton infant aged 6–17 months at baseline, reside in the designated clusters for a minimum of 6 months, and have no intention to relocate during the study period. Exclusion criteria included mothers who were severely ill or unable to communicate, and infants with major congenital anomalies or acute illness at baseline. Infants were enrolled at 6–17 months of age so that, over the 6‐month intervention, the study population spanned 6–23 months, the age range for which the WHO infant and young child feeding indicators (MDD, MMF, MAD) are defined.

The initial sample size was estimated using G Power (version 3.1.5) based on a one‐tailed test, *α* = 0.05, power (1 – *β*) = 0.80, an allocation ratio of 1:1, and an expected effect size of 16% for MAD (Abiyu and Belachew 2020b), yielding a base sample size of 250 participants. To account for clustering, a design effect was applied using the standard formula: DE = 1 + (m – 1) × ICC, where m represents the average cluster size and ICC the intra‐cluster correlation coefficient. An average cluster size of 30 caregivers per cluster and an ICC of 0.02 were assumed based on prior community‐based studies (Shersten and Ziyad Mahfoud 2004), resulting in a design effect of 1.58.

After adjusting for clustering and 20% loss to follow‐up, the final sample size was 474 participants (237 per arm), corresponding to 16 clusters (8 per arm). The number of clusters was determined based on a balance between statistical power requirements and operational feasibility in the pastoral context. The observed cluster size was consistent with these assumptions. A total of 16 clusters were included in the study, with an average of 29.6 caregivers per cluster. Cluster sizes ranged from 10 to 57 participants.

### 2.3 Sampling Techniques, Randomization, and Recruitment

Among the four agro‐pastoral districts, Dubti District was selected by lottery. To facilitate community‐level engagement and ensure logistical feasibility, the Dala defined as culturally recognized clusters were used as the unit of randomization. A Dala is primarily a social and kinship‐based unit rather than a purely geographic or administrative entity. Although Dalas are spatially clustered, they are defined by kinship ties, clan affiliation, and strong social cohesion, including shared decision‐making and mutual support systems. In contrast, administrative units such as villages or garees may include multiple Dalas that are not necessarily socially or familiarly linked. Importantly, all households in the study area belong to a Dala structure, making it an appropriate and comprehensive unit for cluster randomization. Within each Dala, mother–infant pairs served as the units of observation.

**Figure 1:**
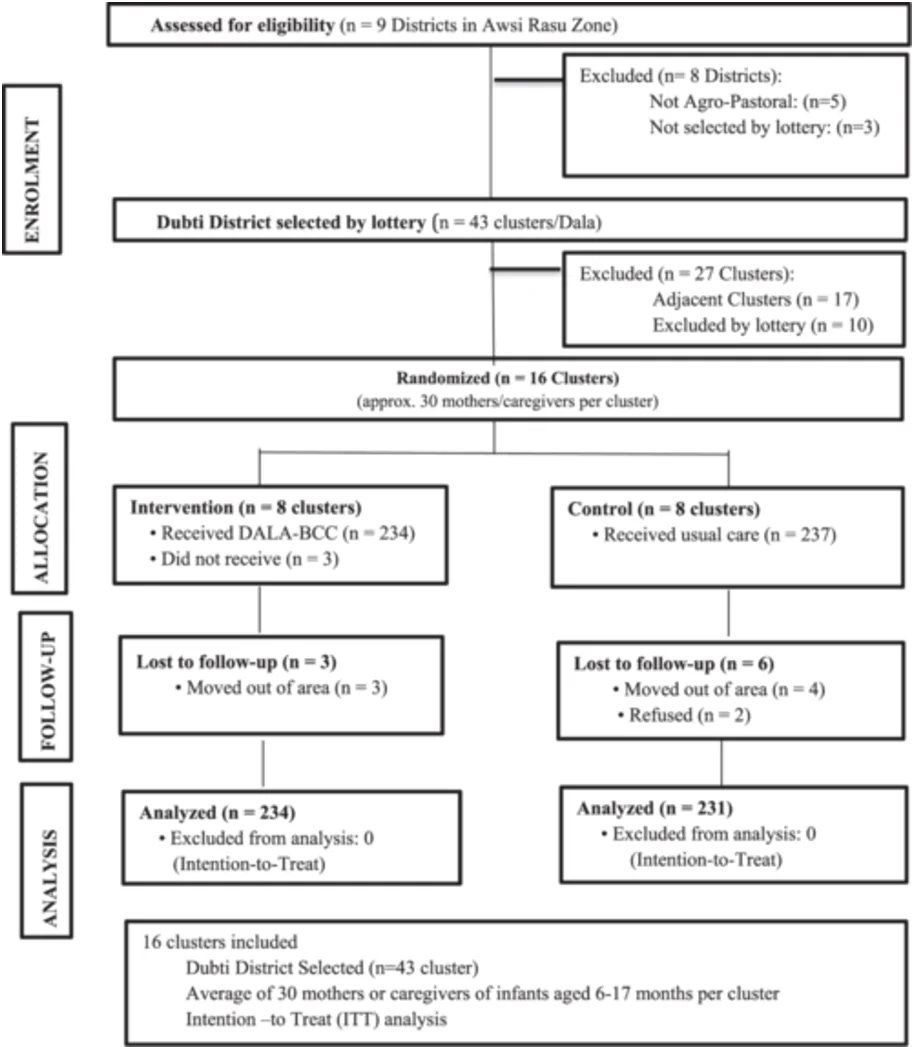
Consolidated Standards of Reporting Trials (CONSORT) flow diagram of participant Recruitment, Randomization, and cluster allocation in the ago‐pastoral community of Afar region, Ethiopia. All “n” values indicate the number of mothers or caregivers of children aged 6–17 months unless otherwise stated.

From the 43 Dala identified in Dubti District, 16 non‐adjacent clusters with no shared boundaries were randomly selected (Figure 1). Of the 43 Dala identified, 17 were excluded for being adjacent to another selected cluster (to minimize contamination between intervention and control conditions), and a further 10 were excluded by simple random sampling to reach the target of 16 clusters, leaving 16 non‐adjacent Dala for inclusion. All 16 Dala leaders and their respective clusters approached for participation agreed to take part; no cluster declined participation, and no cluster withdrew or was lost after randomization. At the individual level, three participants in the intervention arm and six in the control arm were lost to follow‐up between baseline and endline (relocation outside the study area in seven cases and, in two control‐arm cases, refusal to participate in the endline assessment), as detailed in Figure 1. Randomization was performed at the cluster (Dala) level using a computer‐generated random sequence generated using the RAND () function in Microsoft Excel, applying simple randomization without stratification or restriction. The allocation sequence was generated by an independent statistician who was not involved in recruitment, intervention delivery, or outcome assessment. Allocation was concealed until all clusters had been enrolled and baseline data collection was completed. Clusters were allocated in a 1:1 ratio: eight to the intervention arm and eight to the control arm.

Participants were recruited at the household level by trained health extension workers (HEWs) within each selected Dala. HEWs used existing routine records to identify eligible mother–child pairs and conducted household visits to invite them to participate in the study, prior to cluster allocation. During recruitment, mothers were informed that the study was evaluating community‐based approaches to improve IYCF practices, and that assignment to the intervention or control condition would be determined by cluster randomization after baseline data collection. They were clearly informed that participation was voluntary, that they could withdraw at any time without consequence, and that refusal would not affect their access to routine health services. Confidentiality of all information was assured. Written informed consent was obtained from all participants prior to enrollment. The average cluster size was approximately 30 mothers or caregivers of infants aged 6–17 months per Dala. The number of eligible caregiver–child pairs within each Dala was obtained from Health Extension Worker records and varied across clusters.

Participants were selected using proportional allocation across Dalas, followed by simple random sampling within each Dala to ensure representativeness. Consequently, the number of participants per cluster ranged from 10 to 57, reflecting variation in cluster size and proportional allocation. Baseline comparability of clusters between intervention and control arms was assessed descriptively at both cluster and individual levels.

Control‐arm participants were recruited using the same eligibility criteria and household‐level procedures as the intervention arm, and recruitment in both arms was completed before cluster randomization. Within the selected control clusters, trained HEWs used routine records to identify eligible mother–child pairs and visited households to invite participation. Participants in both arms were informed that the study was evaluating community‐based approaches to improve IYCF practices, that participation involved baseline and endline interviews and measurements, and that allocation to the intervention or control condition was determined by cluster randomization; participation was voluntary and written informed consent was obtained prior to enrollment. Participants in the control clusters continued to receive the routine maternal and child health and nutrition services available through the Ethiopian primary healthcare system (“usual care”). These services included IYCF counseling provided by HEWs during facility visits and community outreach, growth monitoring and promotion, immunization, vitamin A supplementation, and management of common childhood illnesses in accordance with national guidelines. No DALA‐BCC group sessions, cooking demonstrations, or home‐based counseling by Dala leaders were provided in the control clusters during the study period, and intervention activities were restricted to the intervention clusters to minimize contamination. To support retention, control‐arm participants were contacted for the scheduled endline assessment using the same follow‐up procedures as the intervention arm; no additional educational activities or incentives were provided to the control clusters, and participants remained enrolled and continued to receive routine healthcare services throughout the follow‐up period.

### 2.4 Blinding

Due to the nature of the community‐based behavioral intervention, blinding of participants and Dala leaders was not feasible. Outcome assessment was blinded, with data collectors and supervisors unaware of cluster allocation. The statistician responsible for data analysis was also blinded to allocation until completion of the primary analyses.

### 2.5 Intervention Package

The DALA‐BCC intervention commenced with the training of Dala leaders—respected and influential figures within pastoralist communities. Eight leaders from the intervention clusters participated in a 7‐day centralized training program focused on key IYCF messages (FMOH 2014), practical cooking demonstrations, and the application of the HBM to guide behavior change strategies. The training employed a participatory, experiential learning approach, incorporating hands‐on practice, demonstrations, facilitated discussions, case studies, role plays, and group exercises. Leaders received culturally tailored visual reference materials and were evaluated through pre‐ and post‐training assessments. Emphasis was placed on small, actionable steps, adult learning principles, and contextually relevant practices to equip leaders with the skills needed to effectively facilitate behavior change among mothers.

Following the leader training, group sessions were conducted for mothers and caregivers. Each Dala leader was assigned 25–30 mothers or caregivers of infants aged 6–17 months and facilitated three structured sessions one at the onset of the intervention and two additional sessions held monthly during the first 3 months. Each session lasted 1.5 to 2 h, allowing sufficient time for cooking demonstrations, interactive counseling, and participatory dialog. The sessions adopted a learner‐centered, discussion‐based approach, incorporating storytelling, experience sharing, negotiation, simulations, demonstrations, and problem‐solving exercises. Culturally and linguistically appropriate posters were used as visual aids to reinforce key complementary feeding messages and build caregiver confidence in adopting recommended practices.

The final component involved home visits, with each Dala leader conducting three visits per household beginning in the third month of the intervention. These visits provided individualized counseling, observation of feeding practices, cooking demonstrations, correction of harmful practices, and feedback on complementary feeding. Fathers and grandmothers were encouraged to participate to support optimal child feeding. Each household received a poster summarizing key messages; the posters were developed with pictorial illustrations, simple graphics, and minimal text in the local language (Afar'af). To accommodate participants with limited literacy, Dala leader explained the content verbally during home visits. All activities were supervised by the researcher with additional oversight from HEWs. The 6‐month intervention was delivered entirely in Afar'af using a multimodal, culturally adapted approach that combined group training, home visits, demonstrations, and printed materials.

The intervention was guided by the HBM, which highlights perceived susceptibility, perceived severity, perceived benefits, perceived barriers, cues to action, and self‐efficacy as key determinants of behavior change. To minimize contamination, non‐adjacent clusters were selected, with equal numbers assigned to intervention and control groups to balance potential variations. All mothers/caregivers in the intervention arm received the same frequency and number of training, delivered with consistent contact duration and supported by a structured training manual (Supplementary file 1).

Intervention fidelity was monitored through structured supervision, observation checklists, and periodic field verification by the principal investigator. The competency of Dala leaders was assessed through pre‐ and post‐training tests. For process evaluation, randomly selected training sessions were observed by a single evaluator to ensure fidelity. Intervention receipt was assessed using structured checklists and interviews to measure mothers'/caregivers' knowledge of child feeding practices and their understanding of the intervention's core content. Intervention enactment was evaluated through direct observation of feeding, nutrition, and health practices for infants and young children.

### 2.6 Data Collection Methods and Procedures

Data were collected using structured, pre‐tested, interviewer‐administered tools that was adapted from the WHO guidelines for assessing IYCF practices, as well as from the Ethiopia Demographic and Health Survey instruments (CSA and ICF 2016; UNICEF & WHO 2021). The tool collected details on household socio‐economic conditions, complementary feeding practices of infants and young children, and mothers' knowledge and attitudes toward child feeding. It also included items based on the HBM, covering perceived susceptibility and severity of malnutrition, perceived benefits and barriers to prevention, cues to action, and self‐efficacy in relation to malnutrition prevention.

To improve accuracy and reduce entry errors, the questionnaire was digitized and deployed on the open data kit (ODK) platform for real‐time data capture. Baseline socio‐demographic information was obtained to establish context, while data on feeding practices, maternal knowledge and attitudes, and HBM constructs were collected at both baseline and end line to track changes and assess the intervention's Impact.

Baseline data were collected at Month 1 prior to intervention implementation. The intervention was delivered over 6 months (Months 2–7). End line data were collected at Month 8.

### 2.7 Outcomes and Measurements

Primary outcomes were MDD, minimum meal frequency (MMF), and MAD, defined according to the WHO and UNICEF Infant and Young Child Feeding indicators (UNICEF and WHO 2021). MDD, MMF, and MAD were derived from children's 24‐h dietary recall across eight food groups: (1) breast milk; (2) grains, roots, tubers, and plantains; (3) pulses, nuts, and seeds; (4) dairy products; (5) flesh foods; (6) eggs; (7) vitamin A–rich fruits and vegetables; and (8) other fruits and vegetables. MDD was defined as consumption of ≥ 5 food groups by children aged 6–23 months. MMF was defined as receiving solid, semi‐solid, or soft foods at the recommended frequency: ≥ 2 times/day for breastfed infants 6–8 months, ≥ 3 times/day for breastfed children 9–23 months, and ≥ 4 times/day for non‐breastfed children 6–23 months (including ≥ 1 solid, semi‐solid, or soft food). MAD included children meeting both MDD and MMF criteria, with non‐breastfed children additionally receiving ≥ 2 milk feeds. Children meeting the criteria were coded as “1,” others as “0.”

Maternal knowledge was measured with six yes/no questions, scored 1 for correct and 0 for incorrect responses. Scores below the mean indicated inadequate knowledge, while scores at or above the mean indicated adequate knowledge (FAO 2016; Fadare et al. 2019). Maternal attitudes were assessed with six statements (“agree,” “disagree,” “don't know”), summed to create an attitude score; scores below the mean reflected a negative attitude, and scores at or above the mean reflected a positive attitude (FAO 2016; Fadare et al. 2019).

Caregivers' perceptions based on the HBM were assessed across six constructs: perceived susceptibility, perceived severity, perceived benefits, perceived barriers, cues to action, and self‐efficacy. Each construct was measured using six binary (yes/no) items, with responses scored as 1 (“Yes”) and 0 (“No”) and summed to generate composite scores ranging from 0 to 6. Higher scores indicate stronger endorsement of each construct (Tariku et al. 2015). Perceived susceptibility reflects caregivers' perceived risk of adverse child health outcomes due to suboptimal feeding, while perceived severity captures the perceived seriousness of such outcomes. Perceived benefits refer to the perceived advantages of recommended feeding practices, whereas perceived barriers represent perceived obstacles to adopting these practices. Cues to action denote factors that prompt behavior change, and self‐efficacy reflects caregivers' confidence in performing recommended feeding practices. Higher scores indicate more favorable perceptions for all constructs except perceived barriers, for which lower scores reflect fewer perceived obstacles and therefore a more favorable status. Primary and secondary outcomes were pre‐specified prior to data analysis in line with CONSORT recommendations.

### 2.8 Data Quality Control

The questionnaire was translated into Afar'af and subsequently back‐translated to ensure accuracy and cultural appropriateness. Data collectors and supervisors received 4 days of intensive training on study protocols and the use of ODK for digital data collection. A pre‐test involving 5% of the sample was conducted, and necessary revisions were made. Data quality was upheld through daily supervisory reviews and field‐level oversight. Blinding procedures were implemented by concealing the specific study objectives from participants, excluding Dala leaders from data collection activities, and randomizing fieldwork schedules to minimize bias. In addition, outcome assessors (data collectors and supervisors) were blinded to cluster allocation, and the statistician remained blinded during the primary analysis phase. Data were collected electronically using ODK, securely backed up, anonymized, and subjected to thorough cleaning prior to analysis, in accordance with strict confidentiality and data protection protocols.

To minimize potential social desirability bias, data collection was conducted by trained independent data collectors who were not involved in the delivery or supervision of the intervention. Dala leaders, who facilitated the BCC sessions and conducted follow‐up visits, were explicitly excluded from all data collection and analysis processes. In addition, caregivers were informed about the confidentiality of their responses and interviews were conducted in a private setting to encourage honest reporting. Furthermore, separation of roles between intervention implementers and outcome assessors was strictly maintained throughout the trial to reduce performance and detection bias.

### 2.9 Data Management and Analysis Methods

Completed questionnaires were reviewed by the principal investigator to ensure completeness before analysis. Data were exported to SPSS version 25 for processing, including the management of missing values, detection of inconsistencies, and correction of errors. Of the 474 mother–child pairs enrolled at baseline, 9 (1.9%) were lost to follow‐up before the endline assessment: three in the intervention arm and six in the control arm. The reasons were relocation outside the study area (*n* = 7) and refusal to participate in the endline assessment (*n* = 2, both in the control arm). Given the very low proportion of missing data, no statistical imputation was undertaken, and analyses were performed using the available observations; the endline and longitudinal GEE analyses were therefore based on 234 participants in the intervention arm and 231 in the control arm, while all 474 enrolled participants were retained in the baseline descriptive comparisons. Descriptive statistics (frequencies, percentages, means, and standard deviations) were generated for socio‐demographic and health‐related characteristics. Baseline characteristics were summarized descriptively by study arm to assess the comparability of the randomized groups, in accordance with the CONSORT extension for cluster randomized trials (Campbell et al. 2012). Formal hypothesis testing of baseline socio‐demographic characteristics was not undertaken because any observed differences are expected to occur by chance following randomization.

The analysis followed an intention‐to‐treat (ITT) approach. Model fit for GEE analyses was assessed using the quasi‐likelihood under the independence model criterion (QIC). Intervention effects on the binary primary outcomes were estimated using GEE models to account for clustering at the Dala level and repeated measurements over time. The binary outcomes (MDD, minimum meal frequency, and MAD) were analyzed using a binomial distribution with a log link. Models included study group, time, and the study group × time interaction, with the interaction term representing the primary estimate of intervention effectiveness (difference‐in‐differences). Models were adjusted for child age, maternal age, maternal education, household wealth index, breastfeeding status, and prior IYCF counseling. Robust sandwich standard errors with an exchangeable working correlation structure were used, and adjusted risk ratios (RRs) with 95% confidence intervals (CIs) are reported. Continuous outcomes (maternal knowledge and attitude scores, and the six HBM construct scores) were summarized as means and standard deviations and compared descriptively between study groups at each time point using independent‐ and paired‐samples *t*‐tests, with mean differences and 95% confidence intervals; these were descriptive analyses and not the primary basis for assessing intervention effectiveness.

All Dala leaders in the study were male, reflecting local leadership norms in the study context. Therefore, subgroup analyses by leader gender were not feasible. In addition, exploratory analyses by other leader characteristics were limited by the small number of clusters.

### 2.10 Ethics Statement

Ethical clearance for this study was obtained from the Institutional Review Board of Jimma University on July 13, 2023 [Ref. JUIH/IRB/555/2023]. Prior to implementation, formal consent was also secured from relevant local authorities. Trained data collectors provided participants with detailed information regarding the study's objectives, procedures, and their rights, including the freedom to decline participation or withdraw at any stage without penalty, as outlined in the participant information sheet. Written informed consent was obtained from all eligible individuals prior to enrollment. The trial was reported in accordance with the CONSORT guidelines for cluster randomized controlled trials (Campbell et al. 2012).

## 3 Results

### 3.1 Baseline Characteristics of Study Participants

A total of 237 mother–infant pairs were evaluated across the intervention and control groups. Children's mean age was 12.76 ± 3.71 months in the intervention group and 12.26 ± 4.17 months in the control group. Overall, baseline socio‐demographic characteristics were well balanced between the intervention and control groups, indicating successful randomization (Table 1). Characteristics of the 16 randomized Dala clusters are summarized in Table 1b. The two arms each comprised 8 clusters with 237 participants; mean cluster size was 29.6 in both arms, although individual cluster sizes varied (intervention range 13–43; control range 10–57), and cluster‐level maternal age and education were comparable between arms. Sex distribution was similar (males: 54.0% vs. 56.5%), most mothers had no formal education (82.3% vs. 84.8%), and a majority of partners were unemployed (78.5% vs. 81.0%). Household wealth and size were also comparable, with high‐income households representing 45.6% versus 38.0%, and households with more than five members accounting for 56.5% versus 54.9%, in the intervention and control groups, respectively.

**Table 1:** Baseline socio‐demographic and maternal characteristics of participants by study group in agro‐pastoral community of afar region, Ethiopia, 2023/24.

Variables	Intervention (*n* = 237) *n* (%)	Control (*n* = 237) *n* (%)
Maternal age		
15–24	79 (33.3%)	65 (27.4%)
25–34	116 (48.9%)	140 (59.1%)
35–49	42 (17.7%)	32 (13.5%)
Child's age		
6–11 months	79 (33.3%)	96 (40.5%)
12–17 months	158 (66.7%)	141 (59.5%)
Sex of index child		
Female	109 (46.0%)	103 (43.5%)
Male	128 (54.0%)	134 (56.5%)
Maternal education		
No formal education	195 (82.3%)	201 (84.8%)
Primary and above	42 (17.7%)	36 (15.2%)
Partner's education		
No formal education	178 (75.1%)	191 (80.6%)
Primary and above	59 (24.9%)	46 (19.4%)
Partner's occupation		
Unemployed	186 (78.5%)	192 (81.0%)
Employed	51 (21.5%)	45 (19.0%)
Household wealth index		
Low income	79 (33.3%)	101 (42.6%)
Middle income	50 (21.1%)	46 (19.4%)
High income	108 (45.6%)	90 (38.0%)
Household size		
≤ 5 members	103 (43.5%)	107 (45.1%)
> 5 members	134 (56.5%)	130 (54.9%)
Received IYCF counseling		
Yes	144 (60.8%)	151 (63.7%)
No	93 (39.2%)	86 (36.3%)
Maternal Attitude		
Unfavorable	102 (43.0%)	94 (39.7%)
Favorable	135 (57.0%)	143 (60.3%)
Maternal Knowledge		
Poor	98 (41.4%)	82 (34.6%)
Good	139 (58.6%)	155 (65.4%)

**Table 1b:** Baseline characteristics of the study clusters (Dala) by study arm in agro‐pastoral community of Afar region, Ethiopia, 2023/24.

Characteristic	Intervention (8 Dala)	Control (8 Dala)
Participants enrolled, *n*	237	237
Mean cluster size (SD)	29.6 (10.8)	29.6 (15.1)
Median cluster size (range)	27.5 (13–43)	28.5 (10–57)
Mean of cluster‐level maternal age, years (SD)	27.6 (1.7)	27.5 (0.7)
Mean of cluster‐level % with no maternal formal education (SD)	81.4 (14.0)	82.4 (10.2)

*Note:* The 16 Dala clusters were allocated 1:1 to the intervention (clusters 1–8) and control (clusters 9–16) arms. SD = standard deviation. Cluster size is the number of participants enrolled per Dala. Cluster‐level characteristics are summarized as the mean (and SD) of the per‐cluster values across the 8 clusters in each arm. Individual‐level baseline characteristics of participants are presented in Table 1.

### 3.2 Changes in Health Belief Model Constructs Following the Intervention

HBM construct scores ranged from 0 to 6, with higher scores indicating more favorable perceptions for all constructs except perceived barriers, where lower scores indicate fewer perceived obstacles.

At baseline, HBM construct scores were comparable between the intervention and control groups (Table 2). At endline, descriptively higher scores were observed in the intervention group for perceived susceptibility, perceived severity, perceived benefits, cues to action, and self‐efficacy, whereas perceived barriers were lower than in the control group. These between‐group comparisons are descriptive summaries; formal evaluation of the intervention effect was based on the adjusted, longitudinal GEE models described in the Statistical Analysis section (Table 6), which account for clustering and repeated measurements and constitute the primary analysis of trial effectiveness.

**Table 2:** Health belief model (HBM) construct scores by study group and assessment period in agro‐pastoral community of Afar region, Ethiopia, 2023/24.

HBM construct	Study period	Intervention (Mean ± SD)	Control (Mean ± SD)	Mean Difference (95% CI)	*p*‐value^†^
Perceived susceptibility	Baseline	2.41 ± 1.07	2.39 ± 1.06	+0.02 (–0.18, +0.21)	—
	Endline	5.95 ± 0.23	2.62 ± 1.23	+3.33 (+3.17, +3.49)	< 0.001
Perceived severity	Baseline	2.89 ± 1.04	2.91 ± 0.85	–0.02 (–0.19, +0.15)	—
	Endline	5.92 ± 0.34	2.62 ± 1.23	+3.30 (+3.14, +3.46)	< 0.001
Perceived benefits	Baseline	2.89 ± 1.11	2.82 ± 1.12	+0.06 (–0.14, +0.26)	—
	Endline	5.79 ± 0.54	2.62 ± 1.23	+3.17 (+2.99, +3.34)	< 0.001
Perceived barriers	Baseline	2.90 ± 0.98	2.86 ± 0.91	+0.04 (–0.13, +0.21)	—
	Endline	1.75 ± 0.93	2.87 ± 0.92	–1.13 (–1.29, –0.96)	< 0.001
Cues to action	Baseline	2.98 ± 1.02	2.94 ± 0.96	+0.04 (–0.14, +0.22)	—
	Endline	5.94 ± 0.30	2.62 ± 1.23	+3.33 (+3.16, +3.49)	< 0.001
Self‐efficacy	Baseline	2.85 ± 1.12	2.89 ± 1.09	–0.03 (–0.23, +0.17)	—
	Endline	5.79 ± 0.54	2.62 ± 1.23	+3.17 (+2.99, +3.34)	< 0.001

*Note:* Values are presented as mean ± standard deviation (SD). Mean differences are calculated as Intervention minus Control. ^†^ *p*‐values represent descriptive between‐group comparisons at endline only. Baseline values are presented descriptively in accordance with CONSORT recommendations for cluster randomized trials. HBM constructs were measured using six binary (yes/no) items per construct, summed to generate scores ranging from 0 to 6. Higher scores indicate stronger endorsement of each construct. For perceived susceptibility, severity, benefits, cues to action, and self‐efficacy, higher scores reflect more favorable perceptions toward recommended infant and young child feeding practices. For perceived barriers, lower scores indicate fewer perceived obstacles and therefore a more favorable status.

### 3.3 Changes in Maternal Knowledge and Attitudes Following the DALA‐BCC Intervention

At baseline, maternal knowledge and attitude scores were comparable between the intervention and control groups. At endline, maternal knowledge was significantly higher in the intervention group (4.26 ± 0.78) than in the control group (3.83 ± 1.08), with a mean difference of 0.43 (95% CI: 0.25–0.60; *p* < 0.001). Similarly, maternal attitude scores were higher in the intervention group (5.70 ± 0.58) compared with the control group (5.17 ± 1.10), with a mean difference of 0.52 (95% CI: 0.36–0.68; *p* < 0.001) (Table 3). These descriptive comparisons are presented to summarize observed group differences in knowledge and attitudes; the primary evaluation of intervention effectiveness on IYCF practices was based on the adjusted GEE models presented in Table 6.

**Table 3:** Maternal knowledge and attitude scores at baseline and endline by study group in the Afar region, Ethiopia (2023/24).

Outcome	Study period	Control (mean ± SD)	Intervention (Mean ± SD)	Mean difference (95% CI)	*p*‐value
Maternal knowledge	Baseline	3.71 ± 0.78	3.57 ± 0.82	—	—
	Endline	3.83 ± 1.08	4.26 ± 0.78	0.43 (0.25–0.60)	< 0.001
Maternal attitude	Baseline	4.68 ± 1.57	4.75 ± 1.39	—	—
	Endline	5.17 ± 1.10	5.70 ± 0.58	0.52 (0.36–0.68)	< 0.001

*Note:* Values are mean ± standard deviation (SD). Mean difference represents intervention minus control at endline. Independent samples *t*‐test was used.

Abbreviation: CI = confidence interval.

### 3.4 Consumption of WHO Food Groups at Endline by Study Group

Table 4 summarizes descriptive endline consumption of WHO food groups by study arm; effect estimates and 95% confidence intervals are presented in the table to quantify the magnitude of these between‐group differences. At endline, consumption of several WHO food groups was higher in the intervention group than in the control group (Table 4). Significant differences were observed for vitamin A–rich fruits and vegetables (53.8% vs. 34.6%, *p* < 0.001), other fruits and vegetables (35.9% vs. 20.3%, *p* < 0.001), and legumes and nuts (58.1% vs. 47.2%, *p* = 0.018).

**Table 4:** Consumption of WHO food groups at end line by study group in the Afar region, Ethiopia (2023/24).

Food Group	Intervention (%)	Control (%)	Difference (%)	RR (95% CI)	*p*‐value
Grains, roots, tubers	97.0	93.1	+3.9	1.04 (1.00–1.09)	0.050
Legumes and nuts	58.1	47.2	+10.9	1.23 (1.04–1.46)	0.018
Dairy products	97.4	94.8	+2.6	1.03 (0.99–1.07)	0.141
Flesh foods	42.3	35.9	+6.4	1.18 (0.94–1.48)	0.159
Eggs	11.1	6.1	+5.0	1.82 (0.98–3.37)	0.052
Vitamin A‐rich fruits & vegetables	53.8	34.6	+19.2	1.55 (1.26–1.92)	< 0.001
Other fruits & vegetables	35.9	20.3	+15.6	1.77 (1.30–2.40)	< 0.001

*Note:* RR = risk ratio (intervention vs. control), with 95% confidence interval.

Consumption of eggs (11.1% vs. 6.1%, *p* = 0.052) and flesh foods (42.3% vs. 35.9%, *p* = 0.159) was higher in the intervention group, although these differences were not statistically significant. Intake of grains, roots and tubers and dairy products exceeded 93% in both groups, with no meaningful between‐group differences.

### 3.5 Impact of the DALA‐BCC on Infant and Young Child Feeding Practices

Baseline proportions of children meeting the core IYCF indicators were similar between the intervention and control groups (Table 5). At endline, descriptively higher proportions of children in intervention clusters met the MDD, minimum meal frequency, and MAD indicators than those in the control clusters: MDD increased to 39.7% versus 24.7%, MMF to 67.9% versus 47.6%, and MAD to 28.6% versus 12.1% (Table 5). Because this study employed a cluster‐randomized design with repeated measurements, the descriptive comparisons presented in Table 5 should be interpreted alongside the adjusted GEE analyses shown in Table 6; the GEE time × study group interaction represents the primary estimate of intervention effectiveness.

**Table 5:** Comparison of core IYCF indicators between intervention and control groups at baseline and end line in agro‐pastoral community of afar region, Ethiopia, 2023/24.

Outcome	Time	Intervention (%)	Control (%)	RR (95% CI)	*p*‐value
Minimum dietary diversity (MDD)	Baseline	21.1	22.8	0.93 (0.66–1.30)	0.657
	Endline	39.7	24.7	1.61 (1.22–2.11)	0.001
Minimum meal frequency (MMF)	Baseline	48.1	45.1	1.07 (0.88–1.29)	0.519
	Endline	67.9	47.6	1.43 (1.22–1.67)	< 0.001
Minimum acceptable diet (MAD)	Baseline	11.8	9.3	1.27 (0.75–2.15)	0.370
	Endline	28.6	12.1	2.36 (1.59–3.52)	< 0.001

*Note:* RR = unadjusted risk ratio with 95% confidence intervals.

**Table 6:** Adjusted effects of the DALA‐BCC intervention on infant and young child feeding indicators using GEE models (intention‐to‐treat analysis), Afar Region, Ethiopia, 2023/24.

Outcome	Baseline comparison (RR, 95% CI)	Time effect (control group) (RR, 95% CI)	Intervention effect (adjusted RR) (95% CI)	*p*‐value
MDD	0.83 (0.54–1.27)	0.63 (0.38–1.04)	2.43 (1.35–4.39)	0.003
MMF	1.12 (0.77–1.61)	1.06 (0.69–1.62)	2.08 (1.25–3.48)	0.005
MAD	1.21 (0.68–2.17)	1.07 (0.54–2.13)	2.35 (1.10–5.04)	0.027

Abbreviations: RR, risk ratio; CI, confidence interval. Intervention effect (difference‐in‐differences) derived from the time × group interaction. Models adjusted for child's age, maternal education, household wealth index, breastfeeding status, maternal age, and IYCF counseling. Robust (sandwich) standard errors accounted for clustering at the Dala level, with an exchangeable working correlation structure; this GEE model constitutes the primary analysis of trial effectiveness.

The following analyses present the primary inferential results of the trial. Intervention effects were estimated using adjusted GEE models, with the study group × time interaction representing the difference‐in‐differences estimate while accounting for clustering and repeated measurements.

### 3.6 Effects of the DALA‐BCC on Core IYCF Indicators: GEE Analysis

GEE models were used to estimate intervention effects, interpreted as the time × group interaction (difference‐in‐differences). No significant baseline differences or changes over time in the control group were observed for MDD, MMF, or MAD. Significant improvements were observed in the intervention group across all outcomes: MDD (RR = 2.43; 95% CI: 1.35–4.39; *p* = 0.003), MMF (RR = 2.08; 95% CI: 1.25–3.48; *p* = 0.005), and MAD (RR = 2.35; 95% CI: 1.10–5.04; *p* = 0.027), indicating improved complementary feeding practices (Table 6).

## 4 Discussion

This cluster randomized controlled trial assessed the effectiveness of the DALA‐BCC intervention in improving key complementary feeding practices. At baseline, the proportions of children meeting IYCF indicators were uniformly low across both study arms. Post‐intervention, significant improvements were observed in the intervention group relative to the control: MDD rose to 39.7% versus 24.7%, MMF increased to 67.9% versus 47.6%, and MAD reached 28.6% compared to 12.1%. These correspond to absolute increases of 15.0, 20.3, and 16.5 percentage points, respectively. After controlling for potential confounders, the multivariable GEE analysis showed that children in intervention clusters had significantly higher probability of achieving optimal complementary feeding practices compared to those in control clusters. Specifically, children in the intervention group were 2.43 times more likely to meet MDD, 2.08 times more likely to achieve MMF, and 2.35 times more likely to attain MAD. These results indicate that structured, community‐based BCC delivered through trusted leaders can meaningfully enhance practices regarding child feeding.

Studies conducted in similar pastoralist settings with comparable interventions are limited, making direct comparisons with the present findings challenging. Nevertheless, our results align with existing evidence indicating that community‐based nutrition behavior change communication (NBCC) interventions can significantly improve IYCF practices across diverse low‐income country contexts (Kuchenbecker 2017; Hoddinott et al. 2018; Abiyu and Belachew 2020a; Fahmida 2020; Rahman et al. 2022; Mondal et al. 2023; Rahman et al. 2023; Han et al. 2024; Verma et al. 2024; Gebretsadik et al. 2025). Prior trials have consistently demonstrated that community‐delivered nutrition education and counseling enhance children's dietary diversity and nutrient adequacy. Evidence from comparable BCC interventions suggests that improvements in complementary feeding practices are typically modest to moderate, often ranging between approximately 5–15 percentage points depending on context, intervention intensity, and delivery platforms. Importantly, the magnitude of effect observed in this study both in absolute percentage point increases and relative risk estimates places these findings at the upper end of, and in some cases exceeding, the range reported in comparable BCC interventions. Specifically, the observed increases of 15.0, 20.3, and 16.5 percentage points in MDD, MMF, and MAD indicate a moderate‐to‐large intervention effect in this setting. The relatively stronger effects observed in this study may be attributable to the culturally adapted and context‐specific design of the intervention, which likely strengthened both its acceptability and effectiveness. Another possible explanation is the timing of outcome assessment: end line data were collected shortly after completion of the 6‐month intervention, capturing relatively short‐term effects, whereas several prior BCC studies assessed outcomes over medium‐ or longer‐term follow‐up periods, during which intervention effects may attenuate. This difference in follow‐up duration may partly explain why the effect sizes observed here are larger than those reported in some earlier trials.

In contrast to the present findings, some studies have reported mixed or non‐significant effects of community‐based interventions. For instance, a cluster‐randomized trial in rural Kenya found no sustained improvements in MDD, MMF, or MAD (Byrd 2018). Similarly, a cluster RCT in Southeast Sulawesi, Indonesia, reported gains in dietary diversity but no significant effect on MMF (Effendy et al. 2020). Such discrepancies may reflect contextual and programmatic differences, including high baseline feeding indicators that limited potential for improvement, insufficient intervention duration or intensity, or reliance on delivery platforms with limited community ownership and sustainability.

This intervention was grounded in the HBM and implemented through the respected Dala leadership structure, strategically enhancing its effectiveness by harnessing community trust, social authority, and cultural alignment. Comparable results have been reported in other trials employing the HBM framework (Vazir et al. 2013; Fahmida 2020), where the active engagement of local leaders and caregivers contributed to improved dietary practices and child health outcomes. Consistent with earlier studies (Rahman et al. 2023; Verma et al. 2024; Ahmed et al. 2025), the current trial also demonstrated significant improvements across key HBM constructs.

In addition, the intervention led to significant improvements in maternal knowledge and attitudes at endline, consistent with the HBM framework. Similar improvements in caregiver knowledge and attitudes following BCC interventions have been reported in previous studies (Fahmida 2020; Vazir et al. 2013, M. Rahman et al. 2023). These findings suggest that the observed improvements in complementary feeding practices may, in part, be mediated through changes in caregivers' knowledge and perceptions. However, as these represent intermediate outcomes, they should be interpreted as supportive evidence of behavioral pathways rather than direct measures of feeding practice change.

These findings suggest that BCC interventions targeting psychosocial determinants such as perceived benefits, perceived barriers, and cues to action can produce measurable and sustained changes in IYCF practices.

The findings have far‐reaching policy implications for improving childhood malnutrition in the agro pastoralist context of the country. This study indicate the value of integrating nutrition BCC into trusted community platforms, guided by behavioral frameworks like the HBM. Such an approach fosters cultural relevance, social legitimacy, and deeper engagement. To advance sustainable improvements in IYCF practices, policymakers should prioritize culturally tailored, community‐led, and multi‐channel strategies that address the psychosocial drivers of behavior and strengthen local ownership.

### 4.1 Strengths and Limitations

This study has several important strengths. The use of a cluster‐randomized controlled design enhances internal validity and supports causal inference. The intervention was culturally grounded and delivered through respected community leaders (Dala), which likely strengthened acceptability, contextual relevance, and intervention fidelity. In addition, the inclusion of HBM constructs enabled examination of potential psychosocial mechanisms underlying changes in IYCF practices, providing insights beyond behavioral outcomes alone.

Several limitations should also be considered. First, the study was conducted in a specific agro‐pastoral context in the Afar Region, which may limit the generalizability of the findings to settings with different socio‐cultural and livelihood characteristics. Second, primary outcomes were based on caregiver self‐reported IYCF practices using 24‐h recall, which is subject to recall error and social desirability bias. This risk may have been amplified in the context of a community‐based intervention in which recommended practices were actively promoted. Although independent data collectors were used to minimize this bias, over‐reporting of desirable behaviors cannot be excluded.

Furthermore, the study did not include objective validation measures, such as direct observation of feeding practices or anthropometric assessments, limiting the ability to independently verify reported behavioral changes. Finally, the relatively small number of clusters may have reduced statistical power and the precision of effect estimates. Although appropriate analytical methods were applied to account for clustering, the limited number of clusters may affect the stability of the estimates. Future studies with a larger number of clusters and inclusion of objective outcome measures are warranted to further strengthen the robustness and generalizability of these findings.

## 5 Conclusion

The findings highlighted that DALA‐BCC intervention significantly enhanced complementary feeding practices in agro‐pastoral communities, increasing the likelihood that children met MDD, meal frequency, and acceptable diet standards. Central to this success was the engagement of culturally respected leaders and the delivery of context‐specific, behaviorally informed messages both of which proved instrumental in influencing caregiver practices. To ensure sustainable impact, Ethiopia's Social and Behavior Change Communication (SBCC) Strategy should integrate pastoralist‐responsive approaches, actively engage traditional leadership structures, and tailor nutrition messaging to reflect local cultural realities.

## Author Contributions

Conceptualization: Anwar Seid Ali, Tefera Belachew, and Kalkidan Hassen Abate. Data curation and Formal analysis: Anwar Seid Ali, Tefera Belachew, Shemsu Kedir and Kalkidan Hassen Abate. Methodology and supervision: Anwar Seid Ali, Tefera Belachew, and Kalkidan Hassen Abate. All authors wrote the paper and Anwar Seid Ali had primary responsibility for the final content. All authors read and approved the final manuscript.

## Conflicts of Interest

The authors declare no conflicts of interest.

## Supporting information


Supporting File

## Data Availability

The data that support the findings of this study are available from the corresponding author upon reasonable request. All data generated or analyzed in this study are included in the article and its supplementary materials. Additional information can be requested from the corresponding author.
