# Supplementary material for: Effect of Dala‐Led Behavior Change Communication on Complementary Feeding Practices in Infants and Young Children: A Cluster‐Randomized Controlled Trial in Agro‐Pastoral Settings of Afar Region, Ethiopia
Source: Matern Child Nutr. 2026 Sep 23;22(3):e70254. doi: 10.1111/mcn.70254 (PMC13599354; doi:10.1111/mcn.70254)
Supplement: Supplementary file 1 [file MCN-22-e70254-s001.docx]

**Supplementary file 1: DALA-BCC Intervention Components, Delivery, and Monitoring among Mothers with Infants and Young Children in the Afar Region, Ethiopia**

| **Contents of the Message / Activity** | **Duration** | **Frequency** | **Strategy of Delivery** | **Compliance Parameter** | **Responsible Person** |
| --- | --- | --- | --- | --- | --- |
| **Training of Dala Leaders** on IYCF, HBM constructs, cooking demonstrations, adult learning principles | 7 days (central training) | Once, before intervention start | Participatory, experiential training: demonstrations, role plays, discussions, hands-on practice, case studies, group exercises; visual aids; pre- and post-tests | % of leaders completing training and passing post-test | Researcher (trainer), supported by HEWs |
| **Key messages for optimal breastfeeding** | 1.5–2 hrs. | One at start + monthly sessions for first 3 months | Group sessions: interactive discussion, storytelling, experience sharing, negotiation, simulations, role play | % of participating mother–infant pairs | Dala leader, supervised by researcher |
| **Key messages on complementary feeding** (timing, frequency, diversity, consistency) | 1.5–2 hrs | One at start + monthly sessions for first 3 months | Cooking demonstrations, visual aids (culturally adapted posters in Afar’af), participatory counseling, group problem-solving | % of participating mother–infant pairs | Dala leader, supervised by researcher |
| **Active feeding techniques** | 1.5–2 hrs | One at start + monthly sessions for first 3 months | Storytelling, simulations, peer-to-peer sharing, demonstrations | % of participating mother–infant pairs | Dala leader, supervised by researcher |
| **Hygienic practices during child feeding** | 1.5–2 hrs | One at start + monthly sessions for first 3 months | Discussion, demonstrations, interactive exercises | % of participating mother–infant pairs | Dala leader, supervised by researcher |
| **Feeding during and after illness** | 1.5–2 hrs | One at start + monthly sessions for first 3 months | Group discussion, culturally adapted posters, problem-solving exercises | % of participating mother–infant pairs | Dala leader, supervised by researcher |
| **Women’s nutrition** (during lactation and pregnancy) | 1.5–2 hrs | One at start + monthly sessions for first 3 months | Group counseling, experience sharing, poster explanation | % of participating mothers | Dala leader, supervised by researcher |
| **Cooking demonstrations & re-demonstrations** | 2 hrs | At each group session | Practical cooking demos, mother re-demonstrations, feedback & correction | % of participating mothers conducting re-demonstration | Dala leader, supervised by researcher |
| **Distribution of print materials** (posters with pictorial illustrations and minimal text in Afar’af) | — | During first training and home visits | Posters/cards explained verbally during sessions and home visits | % of mothers receiving and understanding materials | Dala leader |
| **Home visits** (individualized counseling, observation, family engagement) | ~1 hr per visit | 3 per household (monthly, beginning in month 3) | Observation of feeding, individualized counseling, cooking demonstrations, feedback; verbal explanation of posters for low-literacy mothers; father & grandmother encouraged to participate | % of households visited | Dala leader, supervised by researcher & HEWs |
| **Supervision and monitoring** | Ongoing | Throughout intervention | Researcher oversight, HEWs support, random observation of sessions, structured checklists, mother/caregiver interviews, direct observation of feeding practices | % of sessions observed; % of mothers assessed for knowledge and practice | Researcher & HEWs |
